# BALF metagenomic next-generation sequencing for the diagnosis of pulmonary mycobacterial infection in persons with HIV: a retrospective, diagnostic accuracy study

**DOI:** 10.3389/fmicb.2025.1689997

**Published:** 2025-12-03

**Authors:** Mengjiao Miao, Chenyu Ma, Jinjin Yang, Xihong Yang, Ziyao Liu, Anni Liu, Zheng Qian, You Ge, Yaling Chen, Guoping Yin, Zhiliang Hu

**Affiliations:** 1 School of Public Health of Nanjing Medical University, The Second Hospital of Nanjing, Nanjing, China; 2 Yixing Center for Disease Control and Prevention, Yixing, Jiangsu, China; 3 Department of Infectious Disease, The Second Hospital of Nanjing, Nanjing University of Chinese Medicine, Nanjing, China

**Keywords:** persons with HIV, pulmonary mycobacterial infection, metagenomic next-generation sequencing, bronchoalveolar lavage fluid, co-infections

## Abstract

Severely immunocompromised persons with HIV (PWH) are vulnerable to pulmonary mycobacterial infections (MBI), including *Mycobacterium tuberculosis* (MTB) and non-tuberculous mycobacteria (NTM). This study aimed to assess the effectiveness of metagenomic next-generation sequencing (mNGS) of bronchoalveolar lavage fluid (BALF) in aiding the diagnosis of pulmonary mycobacterial infections in PWH. This study encompassed 146 hospitalized PWH who had a CD4+ T cell count of less than 200 cells/μL. We employed BALF mNGS to pinpoint the causative pathogens of pulmonary infections, with particular focus on pulmonary mycobacterial infections. We evaluated the diagnostic performance of BALF mNGS, and interpreted its clinical significance in detecting mixed infections as appropriate. The median CD4+ T cell count of the participants was 22.5 (IQR: 7.0–63.0) cells/uL. BALF mNGS analysis of 146 severely immunocompromised PWH identified *Mycobacterium tuberculosis* (13.0%) and M. avium complex (7.5%) as the predominant mycobacterial species, with 9.3% (4/43) of mycobacterial infections showing mixed speciation including TB-NTM co-infections or interspecies NTM coinfections. Furthermore, mNGS demonstrated 78.8% sensitivity (95% CI: 62.2%−89.3%) for proven mycobacterial infections, outperforming conventional culture (68.4% vs. 42.1%, *P* < 0.01), though missing 7 proven MBI cases. Finally, among 158 co-detected pathogens, *Pneumocystis jirovecii* (67.1%) and cytomegalovirus (63.0%) were most prevalent, demonstrating co-occurrence rates of 53.5% and 55.8%, respectively in mycobacterial-infected patients. These rates were elevated to 81.2% (*P. jirovecii*) and 65.3% (CMV) in the subset of 101 patients with CD4+ counts <50 cells/μL. The presence of atypical clinical presentations, along with the coexistence of multiple opportunistic pathogens in BALF, complicates the management of pulmonary MBI in PWH. In this context, mNGS has emerged as a highly promising microbiological test that could revolutionize the management of pulmonary MBI in PWH.

## 1 Introduction

In persons with HIV (PWH), pulmonary mycobacterial infections (MBI) represent a significant burden of disease and remain a leading cause of morbidity and mortality, particularly in those with advanced immunosuppression (Moges and Lajore, 2024; Kassaw et al., 2024; Hu et al., 2022). The epidemiology of pulmonary mycobacterial infections in PWH demonstrates that both Mycobacterium tuberculosis (MTB) and non-tuberculous mycobacteria (NTM) can effectively exploit host immunodeficiency, leading to diverse clinical manifestations and heterogeneous treatment outcomes (Llibre et al., 2021). Currently, diagnosing pulmonary MBI in people with HIV remains challenging. Firstly, mycobacterial infections, particularly those caused by NTM, often present with nonspecific clinical manifestations, increasing risks of both missed and misdiagnoses (Nong et al., 2021; Kacprzak et al., 2022; Feng et al., 2025). Secondly, conventional mycobacterial cultures are time-consuming and fail to provide species-level identification for NTM isolates (Luukinen, 2025). Finally, the occurrence of mixed infections, involving multiple pathogens concurrently, further complicates diagnosis decisions (Sheikholeslami et al., 2015). Addressing these challenges requires innovative approaches to improve diagnostic accuracy and facilitate prompt and appropriate management of pulmonary mycobacterial infections in people with HIV.

Recent advancements in metagenomic next-generation sequencing (mNGS) provide a promising solution to the diagnostic challenges posed by pulmonary MBI in PWH. Unlike conventional diagnostic methods that rely on predefined targets, mNGS enables unbiased detection of diverse pathogens in a single assay, making it theoretically capable of identifying all potential pathogens present in a sample. This unbiased approach circumvents the need for specific hypotheses regarding the etiology of infection, allowing for comprehensive pathogen identification (Gu et al., 2019; Miller and Chiu, 2022). This novel technique has demonstrated high sensitivity and specificity in detecting pathogens across various clinical specimens from different infection sites, such as the central nervous system (CNS) and the lungs (Zhu et al., 2022a,b; Fang et al., 2020; Chen et al., 2022; Miao et al., 2018; Yan et al., 2021; Huang et al., 2020). However, available evidence remains limited regarding the role of mNGS in distinguishing pulmonary mycobacterial infections in PWH. The present study aimed to assess the effectiveness of mNGS of bronchoalveolar lavage fluid (BALF) in aiding the diagnosis of pulmonary mycobacterial infections in PWH. Our study primarily focused on MTB and NTM, systematically evaluating the diagnostic performance of BALF mNGS using standardized criteria, with particular emphasis on interpreting polymicrobial infections involving mycobacteria and other co-pathogens.

## 2 Patients and methods

### 2.1 Patients and study design

**Figure 1:**
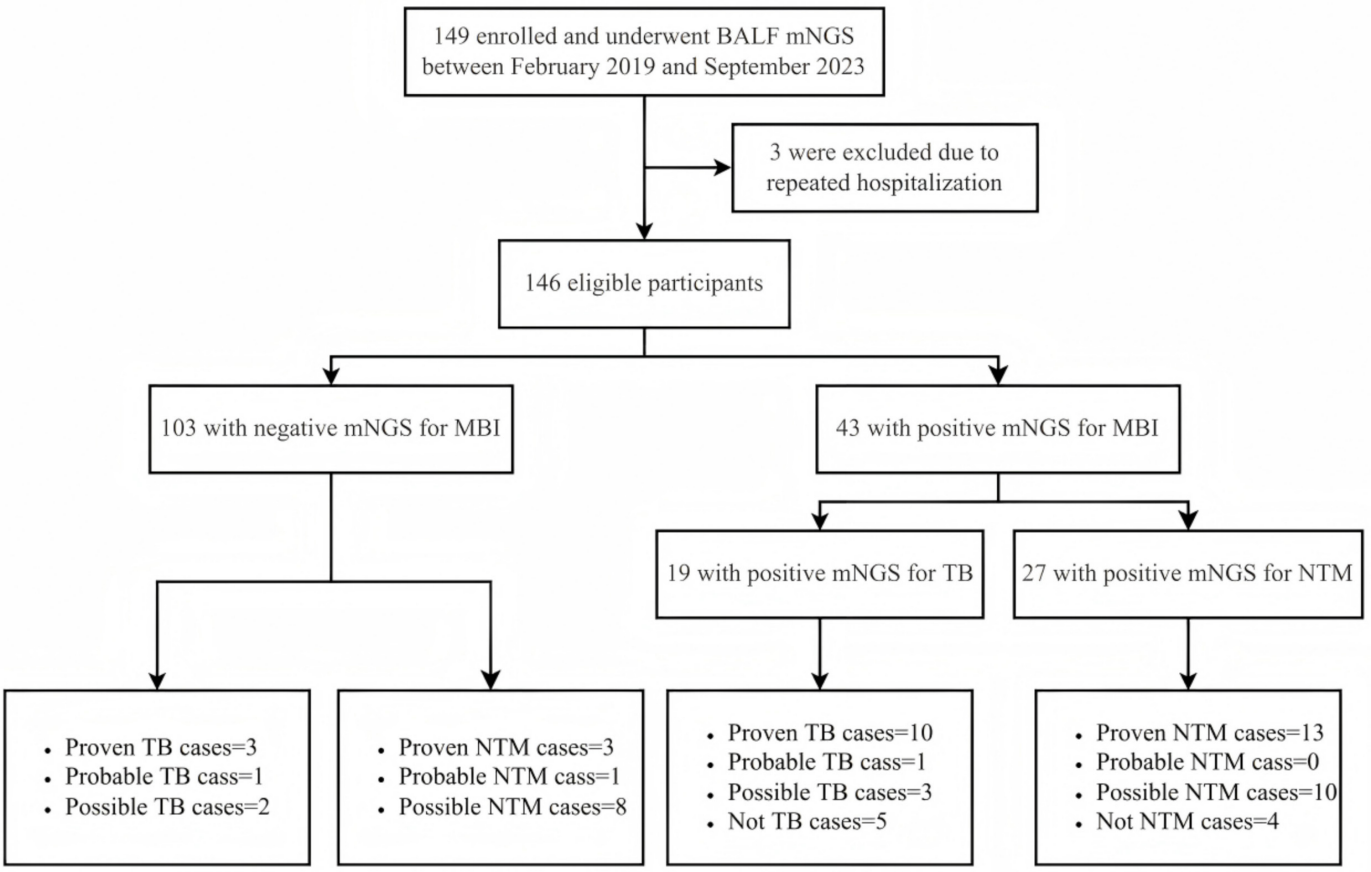
Flowchart of case selection process. The flow diagram illustrates patient inclusion and exclusion throughout the study. A total of 146 PWH who underwent BALF mNGS for suspected pulmonary infection were screened. Final diagnostic classification was based on the CRS for pulmonary MBI, including TB and NTM. PWH, persons with HIV; BALF, bronchoalveolar lavage fluid; mNGS, metagenomic next-generation sequencing; CRS, composite reference standard; MBI, mycobacterial infection; TB, tuberculosis*;* NTM, non-tuberculous mycobacteria.

We identified 149 patients from the Electronic Medical Record (EMR) system of the Second Hospital of Nanjing, China. These patients, who underwent BALF mNGS and had CD4 counts of less than 200 cells/μL, were enrolled in the parent study from February 2019 through September 2023, and 146 (98.0%) were eligible for this analysis (Figure 1). It is important to note that while mNGS was routinely recommended during this period, the final decision to perform the test was subject to patient consent, primarily influenced by factors such as cost considerations. Thus, our study represents a subset of patients who consented to and received the test. Three patients (2.0%) underwent multiple BALF mNGS tests during hospitalization, with only the first result per patient included in the current study, and the others were excluded.

The study institution is a tertiary-care hospital in Jiangsu Province, China, designated as a regional center for infectious diseases and comprehensive HIV management. A standardized diagnostic protocol was in place for all hospitalized PWH who had CD4 counts below 200 cells/μL. Specifically, the protocol included a serum cryptococcal antigen lateral flow assay, an interferon-gamma release assay (IGRA), and blood CMV DNA testing to screen for cryptococcosis, tuberculosis, and cytomegalovirus infection, respectively, as recommended by expert guidelines (Fang et al., 2023; Xu et al., 2020). BALF samples underwent conventional microbiological testing, such as acid-fast staining, bacterial and mycobacterial cultures, Xpert MTB/RIF, and CMV DNA detection in selected samples. Additionally, BALF samples were analyzed using PMSeq-DNA, a commercial mNGS service by BGI-Shenzhen, targeting pathogen DNA sequences (Zhu et al., 2022b).

All clinical data, including demographic information, underlying conditions, laboratory data, mNGS results, treatments, and outcomes, were retrospectively extracted from the hospital's EMR system by two independent investigators using a standardized data collection form. Any discrepancies were adjudicated by a third researcher through re-examination of the original records to ensure accuracy. The compiled data, which integrated mNGS reports with conventional laboratory and clinical findings, formed the unified dataset for all subsequent analyses. The study was approved by the Ethics Committee of the Second Hospital of Nanjing (Approval No: 2024-LS-ky076), the requirement for informed consent was waived by the committee due to the retrospective nature of the study and the use of anonymized data.

### 2.2 Mycobacterial culture

BALF samples (≥5 mL) were processed using the standard protocol of the BACTEC™ MGIT™ 960 mycobacterial detection system. Specimens underwent NALC–NaOH decontamination and were inoculated into MGIT culture tubes supplemented with OADC enrichment and PANTA antibiotic mixture, as recommended by the manufacturer. In parallel, all processed BALF samples were also inoculated onto Lowenstein–Jensen solid medium for quality control, with weekly inspection for colony growth. Positive cultures were confirmed by Ziehl–Neelsen staining before reporting.

### 2.3 PMSeq-DNA extraction via a commercial service

In this study, only one BALF sample was collected per patient, and mNGS was conducted as a single test. All laboratory procedures, from nucleic acid extraction to sequencing, were performed by the commercial service provider BGI-Shenzhen. The work was carried out in strict accordance with their standardized and rigorous PMSeq-DNA protocol, as detailed below: a total of 600 μL of BALF samples were thoroughly mixed with glass beads, then vigorously vortexed at speeds ranging from 2,800 to 3,200 rpm for 30 min. Subsequently, 7.2 μL of lysozyme was added to initiate the cell wall disruption process. Following this, 300 μL of the mixture was transferred to a new 1.5 mL microcentrifuge tube for the extraction of total DNA, utilizing the TIANamp Micro DNA Kit (DP316, Tiangen Biotech). The construction of DNA libraries involved steps such as DNA fragmentation, end-repair, adapter ligation, and PCR amplification. Quality control was performed using the Agilent 2100 system, after which the qualified libraries were pooled. DNA Nanoballs (DNB) were generated and sequenced on the MGISEQ-2000 platform. Sequences of low quality and those aligned with the human reference genome (hg19) were excluded. The refined dataset, after removing low-complexity reads, was subjected to classification by aligning with the Pathogen Metagenomics Database (PMDB), which includes databases of bacteria, fungi, viruses, and parasites. These classification reference databases were sourced from NCBI (ftp://ftp.ncbi.nlm.nih.gov/genomes/), encompassing 5,050 viral taxa whole genome sequences, 10,989 bacterial genomes or scaffolds, 1,179 fungal sequences related to human infections, and 282 parasites linked to human diseases.

### 2.4 Case definition

We applied a composite reference standard (CRS) to pulmonary MBI, pulmonary MTB, and pulmonary NTM infection, categorizing each into three diagnostic confidence levels: “Proven,” “Probable,” and “Possible” (Daley et al., 2020; Miao et al., 2025; World Health Organization, 2024). The detailed criteria are summarized in Table 1, and the corresponding case numbers are presented in Table 2. Proven cases were defined by microbiological confirmation from respiratory samples, meeting the following criteria: (1) Evidence of pulmonary mycobacterial infection: at least one respiratory sample positive for mycobacteria by culture, Xpert MTB/RIF, or other validated microbiological methods; and (2) Species-level identification: for classification into MTB or NTM infection, species identification was required. Cases with pulmonary mycobacterial isolates not identified to species level could be classified as proven MBI, but not further distinguished into MTB or NTM. Probable cases were defined as those without microbiological confirmation from respiratory specimens, but in which mycobacteria were detected in extrapulmonary specimens, or mycobacterial infection was confirmed without species identification, and the expert panel reached diagnostic consensus supporting pulmonary involvement. Possible cases were defined solely based on expert panel consensus, in the absence of any microbiological evidence. Of note, a single positive sputum culture required corroborating histopathological or radiological evidence, along with compatible clinical features, to rule out contamination or colonization. The mNGS results were classified as false positive when mycobacterial sequences were detected by mNGS, but the case did not meet the CRS, and was further adjudicated by a multidisciplinary clinical expert panel as not having active mycobacterial disease.

**Table 1:** Case definitions.

**Definitions**
**1 Proven Cases**
**1.1 Pulmonary** ***Mycobacterium tuberculosis*** **infection (MTB)**
Respiratory samples tested positive by either: (a) Xpert MTB/RIF assay; or (b) culture with species identification as MTB; or (c) evidence of mycobacterial infection (e.g., positive AFB or mycobacterial culture without species identification) combined with confirmed MTB infection from an extrapulmonary site.
**1.2 Pulmonary non-tuberculous mycobacteria infection (NTM)**
(a) Culture with species identification as NTM from at least one BALF, lung tissue, or at least two separate sputum samples; or (b) a single sputum culture positive for NTM species identification, combined with clinical/radiological features compatible with pulmonary NTM, as determined by expert panel consensus; or (c) evidence of mycobacterial infection from respiratory samples (e.g., positive AFB or mycobacterial culture without species identification) combined with confirmed NTM infection from an extrapulmonary site.
**1.3 Pulmonary mycobacterial infection (MBI)**
(a) Meets 1.1 proven MTB or 1.2 proven NTM criteria above; or (b) respiratory samples AFB or mycobacterial culture positive without species identification.
**2 Probable Cases**
**2.1 Pulmonary MTB**
For patients without microbiological evidence of mycobacterial infection in respiratory samples: (a) extrapulmonary specimens positive by Xpert MTB/RIF or culture with species identification as MTB; or (b) extrapulmonary specimens AFB positive or mycobacterial culture positive without species identification, combined with clinical/radiological features compatible with pulmonary MTB, as determined by expert panel consensus. In addition, (c) ^*^cases meeting 1.3(b) for which clinical/radiological features supported MTB after expert review are also classified as probable MTB.
**2.2 Pulmonary NTM**
For patients without microbiological evidence of mycobacterial infection in respiratory samples: (a) extrapulmonary specimens culture positive with species identification as NTM; or (b) extrapulmonary specimens AFB positive or mycobacterial culture positive without species identification, combined with clinical/radiological features compatible with pulmonary NTM, as determined by expert panel consensus. In addition, (c) ^*^cases meeting 1.3(b) for which clinical/radiological features supported NTM after expert review are also classified as probable NTM.
**2.3 Pulmonary MBI**
Any case fulfilling criteria 2.1 (a–b) or 2.2 (a–b).
**3 Possible Cases**
**3.1 Pulmonary MTB**
The diagnosis of pulmonary MTB was based on expert panel consensus without any microbiological evidence.
**3.2 Pulmonary NTM**
The diagnosis of pulmonary NTM was based on expert panel consensus without any microbiological evidence.
**3.3 Pulmonary MBI**
The diagnosis of pulmonary MBI was based on expert panel consensus without any microbiological evidence.

^*^All these cases had clear microbiological evidence of respiratory mycobacterial infection and were therefore classified as proven MBI. However, due to the lack of species identification, they were ultimately classified as MTB or NTM infection based on a comprehensive assessment of clinical and radiological findings, and thus did not meet the criteria for proven pulmonary MTB or NTM infection.

MBI, Mycobacterial infection; AFB, acid-fast bacillus; MTB, Mycobacterium tuberculosis; NTM, non-tuberculous mycobacteria.

**Table 2:** Numbers of pulmonary Mycobacterium tuberculosis infection.

**Criteria** **1. Proven cases**	**Number of patients (n/N)**
**1.1 Pulmonary MTB**	**13/146 (8.9%)**
1.1a	7/146 (4.8%)
1.1b	6/146 (4.1%)
1.1c	0/146 (0.0%)
**1.2 Pulmonary NTM**	**18/146 (12.3%)**
1.2a	18/146 (12.3%)
1.2b	0/146 (0.0%)
1.2c	0/146 (0.0%)
**1.3 Pulmonary MBI**	**33/146 (22.6%)**
1.3a	31/146 (21.2%)
1.3b^*^	2/146 (1.4%)
**2. Probable cases**	
**2.1 Pulmonary MTB**	**3/146 (2.1%)**
2.1a	1/146 (0.7%)
2.1b	0/146 (0.0%)
2.1c	2/146 (1.4%)
**2.2 Pulmonary NTM**	**1/146 (0.7%)**
2.2a	1/146 (0.7%)
2.2b	0/146(0.0%)
2.2c	0/146(0.0%)
**2.3 Pulmonary MBI**	**2/146 (1.4%)**
**3. Possible cases**	
**3.1 Pulmonary MTB**	**5/146 (3.4%)**
**3.2 Pulmonary NTM**	**18/146 (12.3%)**
**3.3 Pulmonary MBI**	**22/146 (15.1%)**

^*^Based on the case definitions of this study, 13 patients were diagnosed with pulmonary MTB, 18 patients were diagnosed with pulmonary NTM infection, while 2 patients could not be clearly classified as either MTB or NTM, but were confirmed to have mycobacterial infections.

MBI, Mycobacterial infection; MTB, Mycobacterium tuberculosis; NTM, non-tuberculous mycobacteria.

### 2.5 Statistical analysis

Categorical variables were shown as frequencies and proportions. Continuous variables were described as medians and interquartile ranges (IQRs). Comparisons between different groups were performed using the chi-square test, Fisher's exact test, or Mann-Whitney U-test when appropriate. Both univariate and multivariable logistic regression analyses were performed to assess factors associated with mNGS positivity. Variables with *p* < 0.10 in univariate analyses or of clinical relevance were included in the multivariable model. Unadjusted and adjusted odds ratios (ORs) with 95% confidence intervals (CIs) are presented. Because culture was included in the CRS and thus no independent gold standard was available, we additionally fitted a two-class latent class model (LCA) based on mNGS and culture results to estimate the sensitivity and specificity of each test without assuming a perfect reference standard. LCA was implemented using the poLCA package in R. We calculated the sensitivity, specificity, positive predictive value (PPV), and negative predictive value (NPV) of mNGS for mycobacterial infection, along with their 95% CIs, using the epi.tests function from the epiR package in R. Sensitivities of different diagnostic methods were compared using McNemar's test. All reported *P*-*values* were 2-sided with the significance level of 0.05. R version 4.4.2 (www.r-project.org) was used for statistical analysis.

## 3 Results

### 3.1 Demographic and clinical characteristics of the study participants

This study included 146 hospitalized PWH who were admitted to the second hospital of Nanjing, China, from February 2019 through September 2023 (Figure 1). Of the 43 participants who obtained a positive mNGS result for MBI, as per the case definition for pulmonary tuberculosis and NTM in this study, 10 (52.6%) were diagnosed with proven tuberculosis, 1 (5.3%) with probable, 3 (15.8%) with possible, and 5 (26.3%) with not tuberculous. As for pulmonary NTM, there were 13 (48.1%) proven cases, 10 (37.0%) possible cases, and 4 (14.8%) non-NTM cases. Notably, among 103 patients with mNGS-negative results for MBI, we identified 3 proven pulmonary tuberculosis cases (2.9%) and 3 proven pulmonary NTM cases (2.9%) that were missed by this method.

Among the 146 participants, 57 (39.0%) were diagnosed with MBI, while 89 (61.0%) were not. The characteristics of the patients included in the study are summarized in Table 3. Patients were generally young, with a median age of 39.0 (IQR: 33.0–49.0) years. Most participants were male (95.9%). A total of 32 (21.9%) patients had a history of smoking. The median CD4+ T cell count was 22.5 (IQR: 7.0–63.0) cells/uL, with 101 (69.2%) patients having a CD4+ T cell count < 50/uL. The study population exhibited elevated HIV viral replication, with an overall median viral load of 5.03 log10 copies/mL (IQR: 3.35–5.53). The majority of patients (84; 57.5%) were not on antiretroviral treatment (ART), 31 (21.2%) had initiated ART within the past 30 days, and another 31 (21.2%) had received ART for more than 30 days. No independent predictors of BALF mNGS positivity were identified in the multivariable analysis (Supplementary Table 1). A non-significant trend toward higher detection was observed among patients with radiological findings compatible with mycobacterial infection (adjusted OR = 1.84, 95% CI: 0.82–4.18).

**Table 3:** Demographic and laboratory characteristics of the patients.

**Characteristics**	**Total *n* = 146**	**Non-MBI** ***n* = 89**	**MBI *n* = 57**	***P-value***
Age	39.00 (33.00, 49.00)	39.00 (33.00, 49.00)	39.00 (32.00, 46.00)	0.76
**Sex**				
Female	6 (4.1)	4 (4.5)	2 (3.5)	1
Male	140 (95.9)	85 (95.5)	55 (96.5)	
Smoking	32 (21.9)	19 (21.3)	13 (22.8)	0.998
Drinking	11 (7.5)	5 (5.6)	6 (10.5)	0.438
Hypertension	8 (5.5)	7 (7.9)	1 (1.8)	0.226
Coronary heart disease	3 (2.1)	1 (1.1)	2 (3.5)	0.694
Cerebrovascular disease	3 (2.1)	3 (3.4)	0 (0.0)	0.422
Diabetes	6 (4.1)	6 (6.7)	0 (0.0)	0.115
CD4 count, cells/μL	22.50 (7.00, 63.00)	29.00 (7.00, 54.00)	22.00 (7.00, 67.00)	0.957
< 50	101 (69.2)	65 (73.0)	36 (63.2)	0.039
50–100	29 (19.9)	12 (13.5)	17 (29.8)	
100–200	16 (11.0)	12 (13.5)	4 (7.0)	
HIV RNA (lg copies/mL)	5.03 (3.35, 5.53)	5.13 (4.68, 5.52)	4.90 (2.59, 5.53)	0.189
< 50 copies/mL	18 (12.3)	9 (10.1)	9 (15.8)	0.344
Missing data	3 (2.1)	1 (1.1)	2 (3.5)	
**ART status**				
Not on ART	84 (57.5)	58 (65.2)	26 (45.6)	0.064
ART ≤ 30 days	31 (21.2)	16 (18.0)	15 (26.3)	
ART > 30 days	31 (21.2)	15 (16.9)	16 (28.1)	
**Blood CMV DNA**				
lg copies/mL	2.72 (2.70, 3.92)	2.88 (2.70, 4.00)	2.70 (2.70, 3.58)	0.196
< 500 copies/mL	69 (47.3)	38 (42.7)	31 (54.4)	0.334
Missing data	8 (5.5)	6 (6.7)	2 (3.5)	
**Blood EBV DNA**				
lg copies/mL	2.70 (2.70, 3.61)	2.70 (2.70, 3.74)	2.70 (2.70, 3.21)	0.056
< 500 copies/mL	81 (55.5)	44 (49.4)	37 (64.9)	0.129
Missing data	12 (8.2)	7 (7.9)	5 (8.8)	

Data were described as median (IQR), n (%). P-values were derived from chi-square test, Fisher's exact test or Mann–Whitney U-test, when appropriate.

MBI, mycobacterial infection; HIV, human immunodeficiency virus; ART, antiretroviral therapy; CMV, cytomegalovirus; EBV, Epstein-barr virus.

### 3.2 Distribution of MBI subspecies by BALF mNGS

**Figure 2:**
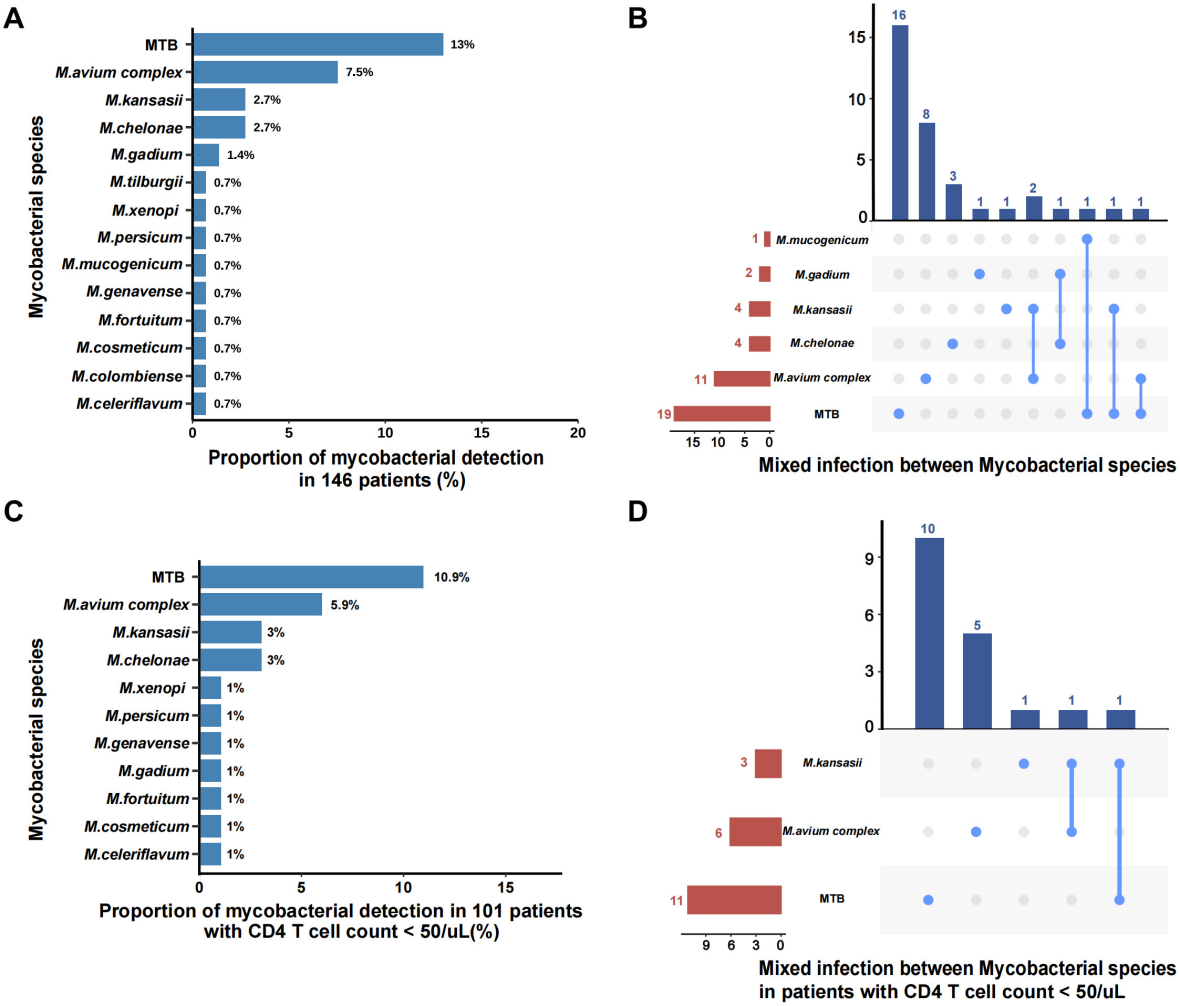
Distribution of MBI subspecies by BALF mNGS. **(A)** All the mycobacterial species detected by BALF mNGS in 146 patients; **(B)** Mixed infection between Mycobacterial species in all patients; **(C)** All the mycobacterial species detected by BALF mNGS in 101 patients with CD4 counts <50 cells/μL; **(D)** Mixed infection between mycobacterial species in patients with CD4 counts < 50 cells/μL. MTB, *Mycobacterium tuberculosis*.

A total of 14 mycobacterial species were detected by BALF mNGS, with the detection rates depicted in Figure 2A. *Mycobacterium tuberculosis* was the most common mycobacterial species (13%; 19/146), followed by *M. avium complex* (7.5%; 11/146), *M. kansasii* (2.7%; 4/146), *M. chelonae* (2.7%; 4/146), and *M. gadium* (1.4%; 2/146).

For the 43 patients who had mNGS results for MBI, simultaneous identification of two mycobacterial species was observed in 6 (14.0%; 6/43) patients (Figure 2B). Notably, BALF mNGS identified 3 cases of tuberculosis-NTM co-infections, among which 2 were subsequently adjudicated as false-positive results (representing single mycobacterial infections) by expert panel review, along with 3 additional confirmed cases of mixed NTM subspecies infections that received diagnostic confirmation from the expert panel. In patients with CD4 counts < 50 cells/μL, the mycobacterial species distribution remained consistent with the overall pattern, with *M. tuberculosis* (10.9%; 11/101), *M. avium complex* (5.9%; 6/101), *M. kansasii* (3%; 3/101), and *M. chelonae* (3%; 3/101) constituting the predominant isolates (Figure 2C). This subgroup analysis identified two noteworthy cases of putative mycobacterial co-infection: one initially suggesting *M. kansasii*–*M. tuberculosis* coexistence that was ultimately classified as singular tuberculosis infection upon expert clinical review, and one confirmed case of authentic *M. kansasii*–*M. avium* complex co-infection that received diagnostic confirmation from the expert panel (Figure 2D).

### 3.3 Diagnostic performance of BALF mNGS for pulmonary mycobacterial infections

**Figure 3:**
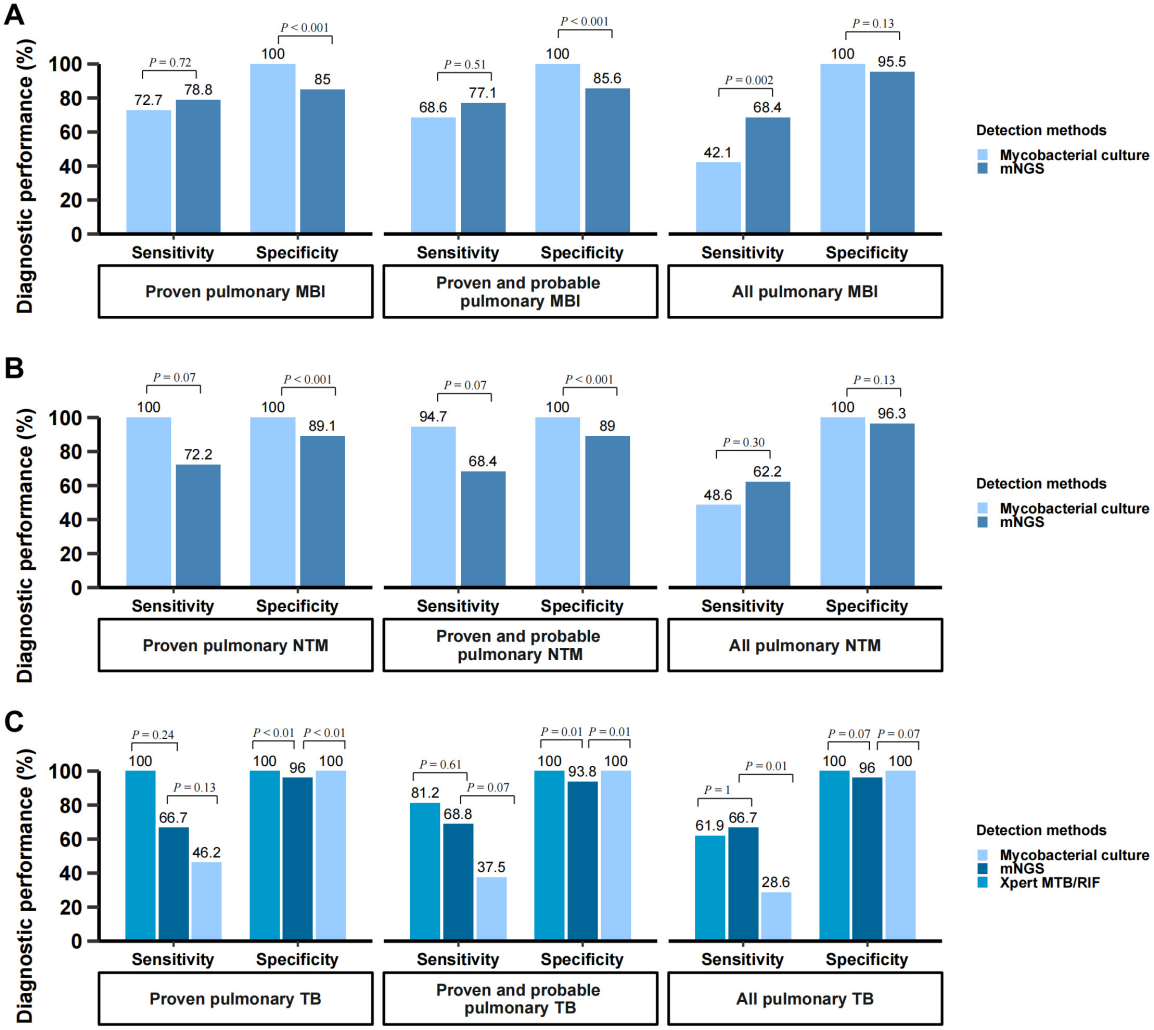
Diagnostic performance of mNGS for pulmonary mycobacterial infection. **(A)** Comparison of diagnostic performance: BALF mNGS vs. mycobacterial culture in diagnosing pulmonary mycobacterial infection; **(B)** Comparison of diagnostic performance: BALF mNGS vs. mycobacterial culture in diagnosing pulmonary NTM infection; **(C)** Comparison of diagnostic performance: BALF mNGS vs. Xpert MTB/RIF assay vs. mycobacterial culture in diagnosing pulmonary TB infection. The vertical axis represents the percentage (%) of each diagnostic metric, including sensitivity and specificity. These metrics were calculated using the composite reference standard. BALF, bronchoalveolar lavage fluid; mNGS, metagenomic next-generation sequencing; MBI, mycobacterial infection; TB, tuberculosis*;* NTM, non-tuberculous mycobacteria.

The diagnostic performance of BALF mNGS for pulmonary mycobacterial infection stratified by different case definitions was summarized in Table 4, and detailed 2 × 2 contingency tables were available in Supplementary Table 6. The sensitivity, specificity, PPV, and NPV of BALF mNGS for diagnosing proven MBI cases were 78.8% (95%CI, 62.2%−89.3%), 85% (95%CI, 77.2%−90.4%), 60.5% (95%CI, 45.6%−73.6%), and 93.2% (95%CI, 86.6%−96.7%), respectively. When further stratified by CD4+ T cell count, the diagnostic performance of BALF mNGS remained comparable between patients with CD4 < 50 cells/μL and those with CD4 50–200 cells/μL. mNGS proved to be more sensitive in diagnosing mycobacterial infections than traditional mycobacterial culture (68.4% vs. 42.1% for all pulmonary mycobacterial infections, *P* < 0.01), although with a potential for false-positive results (Figure 3A). For pulmonary NTM infections, mNGS showed non-inferior sensitivity relative to conventional mycobacterial culture methods, with no statistically significant difference observed between the two diagnostic approaches (Figure 3B). Notably, this diagnostic parity extended to pulmonary tuberculosis detection, where mNGS achieved sensitivity comparable to the Xpert MTB/RIF assay across all case classifications (Figure 3C). Additionally, four patients were mNGS-positive for MBI but did not meet the MBI criteria, and their details were summarized in Supplementary Table 2. Another seven patients with proven MBI were not detected by mNGS; their details were in Supplementary Table 3.

**Table 4:** Diagnostic performance of BALF mNGS for pulmonary mycobacterial infection.

**Pathogens**	**Sensitivity (95% CI; n/N)**	**Specificity** **(95% CI; n/N)**	**PPV (95% CI; n/N)**	**NPV (95% CI; n/N)**
**Pulmonary MBI**				
Proven (n = 33)^*^	78.8 (62.2–89.3; 26/33)	85 (77.2–90.4; 96/113)	60.5 (45.6–73.6; 26/43)	93.2 (86.6–96.7; 96/103)
CD4 < 50 cells/μL	76.2 (54.9–89.4; 16/21)	85 (75.6–91.2; 68/80)	57.1 (39.1–73.5; 16/28)	93.2 (84.9–97; 68/73)
CD4: 50–200 cells/μL	83.3 (55.2–95.3; 10/12)	84.8 (69.1–93.3; 28/33)	66.7 (41.7–84.8; 10/15)	93.3 (78.7–98.2; 28/30)
Proven + probable (*n* = 35)	77.1 (61–87.9; 27/35)	85.6 (77.9–90.9; 95/111)	62.8 (47.9–75.6; 27/43)	92.2 (85.4–96; 95/103)
CD4 < 50 cells/μL	77.3 (56.6–89.9; 17/22)	86.1 (76.8–92; 68/79)	60.7 (42.4–76.4; 17/28)	93.2 (84.9–97; 68/73)
CD4: 50–200 cells/μL	76.9 (49.7-−91.8; 10/13)	84.4 (68.2–93.1; 27/32)	66.7 (41.7–84.8;10/15)	90 (74.4–96.5; 27/30)
Proven + probable + possible (*n* = 57)	68.4 (55.5–79; 39/57)	95.5 (89–98.2; 85/89)	90.7 (78.4–96.3; 39/43)	82.5 (74.1–88.7; 85/103)
CD4 < 50 cells/μL	66.7 (50.3–79.8; 24/36)	93.8 (85.2–97.6; 61/65)	85.7 (68.5–94.3; 24/28)	83.6 (73.4–90.3; 61/73)
CD4: 50–200 cells/μL	71.4 (50–86.2; 15/21)	100 (86.2–100; 24/24)	100 (79.6–100; 15/15)	80 (62.7–90.5; 24/30)
**Pulmonary tuberculosis infection**				
Proven (*n* = 13)	76.9 (49.7–91.8; 10/13)	93.2 (87.6–96.4; 124/133)	52.6 (31.7–72.7; 10/19)	97.6 (93.3–99.2; 124/127)
CD4 < 50 cells/μL	75 (40.9–92.9; 6/8)	94.6 (88–97.7; 88/93)	54.5 (28–78.7; 6/11)	97.8 (92.3–99.4; 88/90)
CD4: 50–200 cells/μL	80 (37.6–96.4; 4/5)	90 (76.9–96; 36/40)	50 (21.5–78.5; 4/8)	97.3 (86.2–99.5; 36/37)
Proven + probable (*n* = 16)	68.8 (44.4–85.8; 11/16)	93.8 (88.3–96.8; 122/130)	57.9 (36.3–76.9; 11/19)	96.1 (91.1–98.3; 122/127)
CD4 < 50 cells/μL	66.7 (35.4–87.9; 6/9)	94.6 (87.9–97.7; 87/92)	54.5 (28–78.7; 6/11)	96.7 (90.7–98.9; 87/90)
CD4: 50–200 cells/μL	71.4 (35.9–91.8; 5/7)	92.1 (79.2–97.3; 35/38)	62.5 (30.6–86.3; 5/8)	94.6 (82.3–98.5; 35/37)
Proven + probable + possible (*n* = 21)	66.7 (45.4–82.8; 14/21)	96 (91–98.3; 120/125)	73.7 (51.2–88.2; 14/19)	94.5 (89.1–97.3; 120/127)
CD4 < 50 cells/μL	63.6 (35.4–84.8; 7/11)	95.6 (89.1–98.3; 86/90)	63.6 (35.4–84.8; 7/11)	95.6 (89.1–98.3; 86/90)
CD4: 50–200 cells/μL	70 (39.7–89.2; 7/10)	97.1 (85.5–99.5; 34/35)	87.5 (52.9–97.8; 7/8)	91.9 (78.7–97.2; 34/37)
**Pulmonary NTM infection**				
Proven (*n* = 18)	72.2 (49.1–87.5; 13/18)	89.1 (82.5–93.4; 114/128)	48.1 (30.7–66; 13/27)	95.8 (90.5–98.2; 114/119)
CD4 < 50 cells/μL	61.5 (35.5–82.3; 8/13)	88.6 (80.3–93.7; 78/88)	44.4 (24.6–66.3; 8/18)	94 (86.7–97.4; 78/83)
CD4: 50–200 cells/μL	100 (56.6–100; 5/5)	90 (76.9–96; 36/40)	55.6 (26.7–81.1; 5/9)	100 (90.4–100; 36/36)
Proven + probable (*n* = 19)	68.4 (46–84.6; 13/19)	89 (82.−93.3; 113/127)	48.1 (30.7–66; 13/27)	95 (89.4–97.7; 113/119)
CD4 < 50 cells/μL	61.5 (35.5–82.3; 8/13)	88.6 (80.3–93.7; 78/88)	44.4 (24.6–66.3; 8/18)	94 (86.7–97.4; 78/83)
CD4: 50–200 cells/μL	83.3 (43.6–97; 5/6)	89.7 (76.4–95.9; 35/39)	55.6 (26.7–81.1; 5/9)	97.2 (85.8–99.5; 35/36)
Proven + probable + possible (*n* = 37)	62.2 (46.1–75.9; 23/37)	96.3 (90.9–98.6; 105/109)	85.2 (67.5–94.1; 23/27)	88.2 (81.2–92.9; 105/119)
CD4 < 50 cells/μL	56 (37.1–73.3; 14/25)	94.7 (87.2–97.9; 72/76)	77.8 (54.8–91; 14/18)	86.7 (77.8–92.4; 72/83)
CD4: 50–200 cells/μL	75 (46.8–91.1; 9/12)	100 (89.6–100;33/33)	100 (70.1–100; 9/9)	91.7 (78.2−97.1; 33/36)

^*^Based on the case definitions of this study, 13 patients were diagnosed with pulmonary MTB, 18 patients were diagnosed with pulmonary NTM infection, while 2 patients could not be clearly classified as either MTB or NTM, but were confirmed to have mycobacterial infections.

BALF, bronchoalveolar lavage fluid; mNGS, metagenomic next generation sequencing; CI, confidence interval; PPV, positive predictive value; NPV, negative predictive value; MBI, mycobacterial infection; NTM, non-tuberculous mycobacteria.

The diagnostic performance of BALF mNGS was further evaluated according to mycobacterial culture time to positivity (TTP) and using a latent class analysis. As shown in Supplementary Table 4, the overall sensitivity of mNGS was 90.9% (20/22) and was similar between fast-growing (TTP ≤ 34.5 days) and slow-growing (TTP > 34.5 days) mycobacterial groups (both 90.9%). In the latent class model, which does not assume a perfect gold standard, mNGS demonstrated higher sensitivity than culture (93.9% vs. 63.2%) with comparable specificity (90.5% vs. 99.3%) (Supplementary Table 5).

### 3.4 Detection of mixed infections of mycobacteria and other pathogens

**Figure 4:**
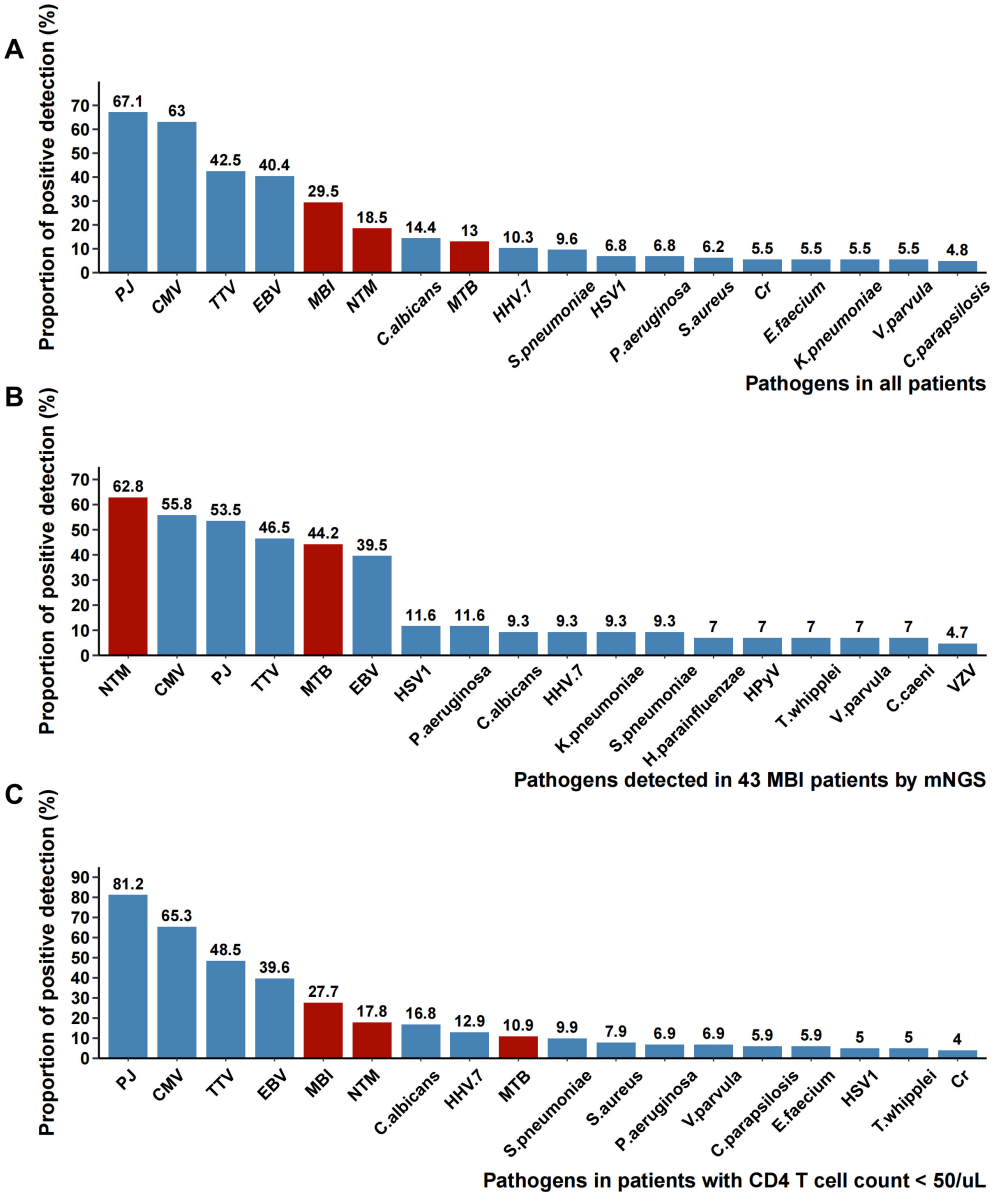
Pathogens detected by BALF mNGS. **(A)** The proportion of each pathogen reported by mNGS in all patients; **(B)** The proportion of each pathogen reported by mNGS in 43 MBI patients; **(C)** The proportion of each pathogen reported by mNGS in patients with CD4 counts <50 cells/μL. PJ, *Pneumocystis jirovecii*; CMV, Cytomegalovirus; TTV, *Torque teno* virus; EBV, *Epstein-Barr* virus; MBI, mycobacterial infection*;* NTM, non-tuberculous mycobacteria; MTB, *Mycobacterium tuberculosis*; HHV.7, *Human herpesvirus-*7; HSV1, *Herpes simplex virus-1*; Cr, *Cryptococcus neoformans*; HPyV, *Human polyomavirus*; VZV, *Varicella-zoster virus*.

A total of 158 pathogens were detected using mNGS. Pathogens present in the top 18 of the mNGS results were illustrated in Figure 4A. The 18 most frequently detected pathogens were *Pneumocystis jirovecii* (67.1%), Cytomegalovirus (63.0%), *Torque teno virus* (42.5%), Epstein-Barr virus (40.4%), mycobacterial infection (29.5%), NTM infection (18.5%), *Candida albicans* (14.4%), Mycobacterium tuberculosis (13%), Human herpesvirus-7 (10.3%), *Streptococcus pneumoniae* (9.6%), Herpes simplex virus-1 (6.8%), *Pseudomonas aeruginosa* (6.8%), *Staphylococcus aureus* (6.2%),*Cryptococcus neoformans* (5.5%)*, Enterococcus faecalis* (5.5%)*, Klebsiella pneumoniae* (5.5%)*, Veillonella parvula* (5.5%)*, Candida parapsilosis* (4.8%). Mycobacterial infection ranked fifth in the pathogen detection rate among all patients, with NTM demonstrating a higher detection rate (18.5%) than MTB (13.0%). In 43 patients detected with MBI, mNGS further revealed co-detections of other pathogens (Figure 4B). Concurrent with mycobacterial infection, the top four co-detecting pathogens were Cytomegalovirus (55.8%), *Pneumocystis jirovecii* (53.5%), *Torque teno virus* (46.5%), and Epstein-Barr virus (39.5%). Among 101 patients with severe immunosuppression (CD4+ T cell count < 50 cells/μL), the pathogen distribution pattern mirrored that of the overall cohort, though with significantly higher detection rates of Pneumocystis jirovecii (81.2% vs 67.1%, *p* = 0.015) (Figure 4C).

## 4 Discussion

Both MTB and NTM infections constitute prevalent opportunistic infections in PWH (Wang et al., 2022; Gopalaswamy et al., 2020). These pathogens present significant diagnostic challenges due to overlapping clinical manifestations and laboratory findings, frequently leading to misclassification in clinical practice (Chiang et al., 2020). The culture-based microbiological methods are commonly utilized in infectious disease for MBI identification. Conventional mycobacterial diagnostics for both MTB and NTM remain constrained by prolonged culture requirements, creating an urgent clinical need for rapid detection methods. Our study demonstrates that mNGS effectively addresses this diagnostic gap by enabling rapid pathogen identification. The study also revealed that the most common mycobacterial species associated with pulmonary infection in this population were MTB, *M. avium complex, M. kansasii, M. chelonae*, and *M. gadium*. Furthermore, we demonstrated that BALF mNGS is more sensitive than traditional mycobacterial culture for diagnosing mycobacterial infection in all pulmonary MBI group, although with a potential for false-positive results. The observed suboptimal detection rate of culture may be attributed to the unique characteristics of the patient population in our tertiary referral center. Many patients had received empirical antibiotic therapy at primary care institutions prior to referral, which may have substantially reduced viable bacterial loads and consequently diminished culture positivity rates.

Establishing the etiological diagnosis of pulmonary infections in PWH is not easily achieved until the emergence of high-throughput molecular methods based on next-generation sequencing. Our current study demonstrates that all clinically prevalent pathogens can be identified by mNGS. Importantly, 94.5% of BALF samples detected PJ, CMV, or MBI (MTB or NTM). The data demonstrated a significantly higher detection rate of *Pneumocystis jirovecii* in hospitalized PWH with CD4 counts below 200 cells/μL (67.1%), which further escalated to 81.2% among those with more severe immunosuppression (CD4 < 50 cells/μL). These findings suggest that for late-presenting PWH with suspected pulmonary infection, initial empirical antimicrobial therapy should prioritize these key opportunistic pathogens, particularly PJ, CMV, and MBI.

Clinicians caring for those patients should also be alarmed about the possible mixed pulmonary infections. The vast majority of BALF samples contained at least one of these pathogens, with approximately 60% of samples containing multiple pathogens. Our findings demonstrate that mNGS successfully detected mixed mycobacterial species infections in 4 cases (9.3%, 4/43), enabling pathogen-directed antimicrobial therapy. This diagnostic capability is particularly valuable given that differentiation of mixed mycobacterial infections remains a significant clinical challenge in routine practice. Among 43 patients diagnosed with MBI via mNGS, CMV emerged as the most prevalent co-pathogen. The observation is consistent with previous reports in the literature (Fang et al., 2025; Kua et al., 2023). This finding underscores the clinical imperative to consider potential CMV-Mycobacterium coinfection when either pathogen is detected in PWH.

While our study confirms the high sensitivity of BALF mNGS for detecting pulmonary NTM, clinical interpretation of positive results requires careful assessment, particularly in PWH or other severely immunocompromised patients. Although earlier intervention may be justified in these vulnerable individuals due to the risk of rapid disease progression, the diagnosis of NTM pulmonary disease must comply with established guidelines, which require concordant clinical and radiological findings together with microbiological confirmation—typically through repeated isolation of NTM from independent respiratory specimens (Daley et al., 2020; Nelson et al., 2024; Griffith et al., 2007). This process often leads to a prolonged diagnostic timeline. In this context, a positive BALF mNGS result should be regarded as an important clue warranting further microbiological verification. It is also important to note that the risk of false-positive results due to environmental or laboratory contamination cannot be overlooked, especially for ubiquitous microorganisms like NTM. Consequently, laboratories must implement stringent measures—such as physical separation of pre- and post-amplification areas, routine surface decontamination, and the inclusion of negative controls in each sequencing run—to minimize this risk. In summary, the core value of mNGS in this context lies not in replacing conventional methods, but in its capacity to rapidly narrow the differential diagnosis, guide subsequent investigations, and thereby accelerate the overall precision diagnostic workflow.

Our findings are generally consistent with previous studies evaluating the diagnostic performance of mNGS in pulmonary infections. Xu et al. (2023) found that in non-immunocompromised patients with lower respiratory tract infections, BALF mNGS demonstrated an overall superior detection rate compared to conventional methods. Similarly, Chen et al. (2022) demonstrated that mNGS exhibited a sensitivity of approximately 78% for various respiratory pathogens, with a notably high positive detection rate of 92% for pathogens in BALF from critically ill or immunocompromised patients. Correspondingly, Zinter et al. (2019) reported that in immunocompromised children, mNGS not only revealed a diverse pulmonary microbiome comprising bacteria, fungi, and viruses in the lower respiratory tract but also identified potential pathogens in half of the samples with negative conventional test results. Sun et al. (2024) highlighted a higher detection rate of Mycobacterium by mNGS in PWH compared to non-HIV groups. Importantly, our study extends this body of evidence by demonstrating that BALF mNGS maintains high diagnostic sensitivity for mycobacterial infections in PWH, even under conditions of severe immunosuppression. Collectively, these findings reinforce the potential of mNGS as a complementary and increasingly indispensable tool for the comprehensive etiological evaluation of pulmonary infections in immunocompromised hosts.

At present, the substantial cost and bioinformatic demands of mNGS limit its widespread use compared to conventional culture. Our study, conducted in Nanjing (an economically developed city in Eastern China), reflects a resource-advantaged setting; however, routine mNGS implementation remains impractical in many resource-limited regions. A promising future model may involve regional third-party sequencing centers that consolidate testing to reduce unit costs and share bioinformatics expertise. Such a centralized approach could enhance the accessibility of mNGS, transforming it from an advanced assay into a practical tool with broader public health value.

Our study has several limitations. First, as a retrospective investigation, we were unable to perform follow-up evaluations on four patients whose mNGS results detected MBI but did not meet the predefined MBI diagnostic criteria. Consequently, the potential for subsequent development of clinically significant mycobacterial infections in these individuals remains undetermined. Secondly, as a single-center retrospective study conducted in a tertiary referral hospital, our findings may reflect institutional or regional characteristics and should be validated in multicenter, prospective cohorts to further establish the diagnostic accuracy of mNGS for MBI. Third, smear grade data were incomplete for a subset of patients, limiting the ability to stratify diagnostic sensitivity by bacterial burden. Finally, as the study population was predominantly male (95.9%), potential gender bias may limit the generalizability of our findings. The clinical interpretation of mNGS results—particularly distinguishing true pathogenic infection from microbial colonization in severely immunocompromised PWH—also warrants further investigation through rigorously designed prospective studies.

## 5 Conclusion

In summary, our study delineates the epidemiological profile of mycobacterial species distribution and concurrent respiratory pathogens in PWH through BALF mNGS. BALF mNGS exhibits superior diagnostic performance for mycobacterial infections while simultaneously enabling rapid identification of polymicrobial co-detections. These findings establish mNGS as a transformative diagnostic platform that could revolutionize the management of pulmonary MBI in PWH.

## Data Availability

The raw data supporting the conclusions of this article will be made available by the authors, without undue reservation.
