# Supplementary material for: BALF metagenomic next-generation sequencing for the diagnosis of pulmonary mycobacterial infection in persons with HIV: a retrospective, diagnostic accuracy study
Source: Front Microbiol. 2025 Dec 3;16:1689997. doi: 10.3389/fmicb.2025.1689997 (PMC12708606; doi:10.3389/fmicb.2025.1689997)
Supplement: Supplementary file 1 [file Table_1.docx]

**Supplemental Table 1**：Univariate and multivariable logistic regression analysis of factors associated with BALF mNGS positivity for mycobacterial infection in persons with HIV

| **Variable** |  | **Unadjusted OR (95% CI)** | ***p*-value** | **Adjusted OR (95% CI)** | ***p*-value** |
| --- | --- | --- | --- | --- | --- |
| CD4 count, cells/μL | <50 | 1 |  | 1 |  |
|  | ≥50 | 1.58 (0.77–3.23) | 0.209 | 0.59  (0.19–1.67) | 0.336 |
| HIV RNA (log10 copies/mL) |  | 0.82 (0.66–1.01) | 0.059 | 0.74  (0.50–1.06) | 0.115 |
| ART | ≤30 days | 1 |  | 1 |  |
|  | >30 days | 1.14 (0.42–3.11) | 0.800 | 0.67  (0.19–2.28) | 0.523 |
|  | Not on ART | 0.48 (0.20–1.11) | 0.086 | 0.79  (0.27–2.47) | 0.671 |
| CT findings suggestive of MBI |  | 2.94 (1.48–5.93) | 0.002 | 1.84  (0.82–4.18) | 0.141 |
| Age,years |  | 1.00 (0.97–1.03) | 0.852 | 1.00  (0.97–1.04) | 0.892 |
| Sex | Female | 1 |  | 1 |  |
|  | Male | 1.29 (0.24–9.56) | 0.770 | 0.58  (0.10–4.61) | 0.558 |

Unadjusted ORs were derived from univariate logistic regression. Adjusted ORs were obtained from multivariable logistic regression models including all listed variables.

Abbreviations: BALF, bronchoalveolar lavage fluid; mNGS, metagenomic next-generation sequencing; MBI, mycobacterial infection; ART, antiretroviral therapy.
