# Supplementary material for: BALF metagenomic next-generation sequencing for the diagnosis of pulmonary mycobacterial infection in persons with HIV: a retrospective, diagnostic accuracy study
Source: Front Microbiol. 2025 Dec 3;16:1689997. doi: 10.3389/fmicb.2025.1689997 (PMC12708606; doi:10.3389/fmicb.2025.1689997)
Supplement: Supplementary file 2 [file Table_2.docx]

**Supplemental Table 2** Characteristics of the 4 patients with MBI detected by mNGS but not meeting MBI criteria

| Pts | 1 | 2 | 3 | 4 |
| --- | --- | --- | --- | --- |
| Age, years | 35-40 | 25-30 | 45-50 | 55-60 |
| BMI | 18.5-23.9 | 18.5-23.9 | <18.5 | 18.5-23.9 |
| CD4 count, cells/μL | 42 | 6 | 7 | 45 |
| Plasma HIV load, copies/ml | 253000 | 328000 | NA | 360000 |
| ART status | not on ART | not on ART | ≤30 days | ≤30 days |
| Blood CMV DNA, copies/ml | 0 | 0 | 170000 | 10000 |
| Blood EBV DNA, copies/ml | 5710 | 0 | 0 | 0 |
| TB mNGS reads | 0 | 4 | 1 | 0 |
| M.fortuitum mNGS reads | 1 | 0 | 0 | 0 |
| M.chelonae mNGS reads | 0 | 0 | 0 | 2 |
| CMV mNGS reads | 3 | 0 | 7339 | 51 |
| EBVmNGS reads | 0 | 0 | 0 | 0 |
| PJ mNGS reads | 956 | 555072 | 1766 | 8 |
| TTV mNGS reads | 0 | 0 | 0 | 21 |
| HHV-7 mNGS reads | 0 | 6 | 0 | 0 |

Abbreviations: BMI, body mass index; HIV, human immunodeficiency virus; ART, antiretroviral therapy; CMV, *cytomegalovirus*; EBV, *Epstein-barr virus*; TB, tuberculosis; mNGS, metagenomic next generation sequencing; PJ, *Pneumocystis jirovecii*; TTV, *Tacaribe virus*; HHV-7, *Human Herpesvirus 7*.
