# Supplementary material for: BALF metagenomic next-generation sequencing for the diagnosis of pulmonary mycobacterial infection in persons with HIV: a retrospective, diagnostic accuracy study
Source: Front Microbiol. 2025 Dec 3;16:1689997. doi: 10.3389/fmicb.2025.1689997 (PMC12708606; doi:10.3389/fmicb.2025.1689997)
Supplement: Supplementary file 3 [file Table_3.docx]

**Supplemental Table 3** Characteristics of 7 patients with proven MBI but negative mNGS results

| Pts | 1 | 2 | 3 | 4 | 5 | 6 | 7 |
| --- | --- | --- | --- | --- | --- | --- | --- |
| Age, years | 40-45 | 30-35 | 30-35 | 35-40 | 30-35 | 25-30 | 35-40 |
| BMI | 24-28 | 18.5-23.9 | 18.5-23.9 | 18.5-23.9 | 18.5-23.9 | 18.5-23.9 | 18.5-23.9 |
| CD4 count, cells/μL | 70 | 6 | 4 | 159 | 1 | 8 | 20 |
| Plasma HIV load, copies/ml | 360000 | 95800 | 1540000 | 2790 | 1290000 | 285000 | 606000 |
| ART status | > 30 days | not on ART | not on ART | > 30 days | not on ART | not on ART | ≤30 days |
| Blood CMV DNA, copies/ml | NA | 0 | 2800 | 0 | 0 | 4000 | 26000 |
| Blood EBV DNA, copies/ml | 0 | 0 | 9800 | 0 | 0 | 0 | NA |
| Extrapulmonary MBC | - | - | + | - | - | + | - |
| Sputum MBC | - | - | - | + | - | + | + |
| BALF xpert | - | + | - | + | + | - | - |
| BALF MBC | - | - | + | - | - | + | - |
| Lung tissue MBC | NA | NA | NA | NA | NA | NA | + |
| Lung tissue T-spot | + | - | - | - | - | - | - |
| MBI treatment | + | + | + | + | + | + | + |
| CMV mNGS reads | 0 | 1355 | 49 | 13 | 62 | 12 | 15 |
| PJ mNGS reads | 0 | 9 | 2775616 | 0 | 1368 | 0 | 72 |
| Cr mNGS reads | 0 | 84 | 0 | 0 | 0 | 0 | 0 |
| EBV mNGS reads | 0 | 1098 | 0 | 0 | 0 | 0 | 7 |
| TTV mNGS reads | 0 | 3839 | 53 | 6 | 665 | 0 | 34 |
| HHV-7 mNGS reads | 0 | 0 | 6 | 0 | 0 | 0 | 0 |

Abbreviations: BMI, body mass index; HIV, human immunodeficiency virus; ART, antiretroviral therapy; CMV, *cytomegalovirus*; EBV, *Epstein-barr virus*; MBC, Mycobacterium culture; MBI, mycobacterial infection; BALF, bronchoalveolar lavage fluid; mNGS, metagenomic next generation sequencing; PJ, *Pneumocystis jirovecii*; Cr, cryptococcus; TTV, *Tacaribe virus*; HHV-7, *Human Herpesvirus 7*.
