# Supplementary material for: BALF metagenomic next-generation sequencing for the diagnosis of pulmonary mycobacterial infection in persons with HIV: a retrospective, diagnostic accuracy study
Source: Front Microbiol. 2025 Dec 3;16:1689997. doi: 10.3389/fmicb.2025.1689997 (PMC12708606; doi:10.3389/fmicb.2025.1689997)
Supplement: Supplementary file 4 [file Table_4.docx]

| Group | No. of Cases | TTP Range (days) | No. of mNGS-positive Cases | Sensitivity of mNGS (%) |
| --- | --- | --- | --- | --- |
| All BALF culture-positive cases | 22 | 20-57 | 20 | 90.9% |
| Fast growth group (TTP ≤ 34.5 days) | 11 | 20-34 | 10 | 90.9% |
| Slow growth group (TTP > 34.5 days) | 11 | 35-57 | 10 | 90.9% |

**Supplemental Table 4** Diagnostic performance of BALF mNGS stratified by BALF mycobacterial culture time to positivity (TTP) (n=22 culture-positive patients)

Groups were stratified based on the median culture time to positivity .A positive mNGS result was defined as the detection of either MTB or NTM.

Abbreviations: BALF, bronchoalveolar lavage fluid; mNGS, metagenomic next generation sequencing; TTP, time to positivity; MTB, Mycobacterium tuberculosis; NTM, nontuberculous mycobacteria.
