# Supplementary material for: BALF metagenomic next-generation sequencing for the diagnosis of pulmonary mycobacterial infection in persons with HIV: a retrospective, diagnostic accuracy study
Source: Front Microbiol. 2025 Dec 3;16:1689997. doi: 10.3389/fmicb.2025.1689997 (PMC12708606; doi:10.3389/fmicb.2025.1689997)
Supplement: Supplementary file 5 [file Table_5.docx]

**Supplemental Table 5:**Diagnostic Performance Based on Latent Class Analysis

|  | BALF mNGS | BALF mycobacterial culture |
| --- | --- | --- |
| Sensitivity, % | 93.9% | 63.2% |
| Specificity, % | 90.5% | 99.3% |
| PPV, % | 47.6% | 90.9% |
| NPV, % | 99.2% | 95.0% |
| Prevalence, % | 23.5% | 23.5% |
| N | 143 | 143 |
| Goodness-of-fit | AIC=260.93 | BIC=275.75 |

This analysis was based on a latent class model, which does not presume a perfect gold standard and allows for the simultaneous assessment of the diagnostic accuracy of both tests. The analysis included 143 patients who had valid results from both BALF mNGS and mycobacterial culture. The model goodness-of-fit indices indicate a well-fitted model. The PPV and NPV were calculated based on the model-estimated disease prevalence of 23.5%.

Abbreviations: BALF, bronchoalveolar lavage fluid; mNGS, metagenomic next-generation sequencing; PPV, positive predictive value; NPV, negative predictive value; AIC, Akaike information criterion; BIC, Bayesian information criterion.
