# Supplementary material for: BALF metagenomic next-generation sequencing for the diagnosis of pulmonary mycobacterial infection in persons with HIV: a retrospective, diagnostic accuracy study
Source: Front Microbiol. 2025 Dec 3;16:1689997. doi: 10.3389/fmicb.2025.1689997 (PMC12708606; doi:10.3389/fmicb.2025.1689997)
Supplement: Supplementary file 6 [file Table_6.docx]

**Supplemental Table 6** 2x2 contingency table for the diagnosis of pulmonary mycobacterial infection (MBI) using metagenomic next-generation sequencing (mNGS) against the composite reference standard (CRS) for proven cases

|  | CRS:  Positive | CRS:  Negative | Total​ |
| --- | --- | --- | --- |
| mNGS: Positive​ | 26 | 17 | 43 |
| mNGS: Negative | 7 | 96 | 103 |
| **Total** | 33 | 113 | 146 |

**Supplemental Table 7** 2x2 contingency table for the diagnosis of pulmonary mycobacterial infection (MBI) using metagenomic next-generation sequencing (mNGS) against the composite reference standard (CRS) for proven+probable cases

|  | CRS:  Positive | CRS:  Negative | Total​ |
| --- | --- | --- | --- |
| mNGS: Positive​ | 27 | 16 | 43 |
| mNGS: Negative | 8 | 95 | 103 |
| **Total** | 35 | 111 | 146 |

**Supplemental Table 8** 2x2 contingency table for the diagnosis of pulmonary mycobacterial infection (MBI) using metagenomic next-generation sequencing (mNGS) against the composite reference standard (CRS) for proven+probable+possible cases

|  | CRS:  Positive | CRS:  Negative | Total​ |
| --- | --- | --- | --- |
| mNGS: Positive​ | 39 | 4 | 43 |
| mNGS: Negative | 18 | 85 | 103 |
| **Total** | 57 | 89 | 146 |

**Supplemental Table 9** 2x2 contingency table for the diagnosis of pulmonary Mycobacterium tuberculosis infection (MTB) using metagenomic next-generation sequencing (mNGS) against the composite reference standard (CRS) for proven cases

|  | CRS:  Positive | CRS:  Negative | Total​ |
| --- | --- | --- | --- |
| mNGS: Positive​ | 10 | 9 | 19 |
| mNGS: Negative | 3 | 124 | 127 |
| **Total** | 13 | 133 | 146 |

**Supplemental Table 10** 2x2 contingency table for the diagnosis of pulmonary Mycobacterium tuberculosis infection (MTB) using metagenomic next-generation sequencing (mNGS) against the composite reference standard (CRS) for proven+probable cases

|  | CRS:  Positive | CRS:  Negative | Total​ |
| --- | --- | --- | --- |
| mNGS: Positive​ | 11 | 8 | 19 |
| mNGS: Negative | 5 | 122 | 127 |
| **Total** | 16 | 130 | 146 |

**Supplemental Table 11** 2x2 contingency table for the diagnosis of pulmonary Mycobacterium tuberculosis infection (MTB) using metagenomic next-generation sequencing (mNGS) against the composite reference standard (CRS) for proven+probable+possible cases

|  | CRS:  Positive | CRS:  Negative | Total​ |
| --- | --- | --- | --- |
| mNGS: Positive​ | 14 | 5 | 19 |
| mNGS: Negative | 7 | 120 | 127 |
| **Total** | 21 | 125 | 146 |

**Supplemental Table 12** 2x2 contingency table for the diagnosis of pulmonary non-tuberculous mycobacteria infection (NTM) using metagenomic next-generation sequencing (mNGS) against the composite reference standard (CRS) for proven cases

|  | CRS:  Positive | CRS:  Negative | Total​ |
| --- | --- | --- | --- |
| mNGS: Positive​ | 13 | 14 | 27 |
| mNGS: Negative | 5 | 114 | 119 |
| **Total** | 18 | 128 | 146 |

**Supplemental Table 13** 2x2 contingency table for the diagnosis of pulmonary non-tuberculous mycobacteria infection (NTM) using metagenomic next-generation sequencing (mNGS) against the composite reference standard (CRS) for proven+probable cases

|  | CRS:  Positive | CRS:  Negative | Total​ |
| --- | --- | --- | --- |
| mNGS: Positive​ | 13 | 14 | 27 |
| mNGS: Negative | 6 | 113 | 119 |
| **Total** | 19 | 127 | 146 |

**Supplemental Table 14** 2x2 contingency table for the diagnosis of pulmonary non-tuberculous mycobacteria infection (NTM) using metagenomic next-generation sequencing (mNGS) against the composite reference standard (CRS) for proven+probable+possible cases

|  | CRS:  Positive | CRS:  Negative | Total​ |
| --- | --- | --- | --- |
| mNGS: Positive​ | 23 | 4 | 27 |
| mNGS: Negative | 14 | 105 | 119 |
| **Total** | 37 | 109 | 146 |
